# Neuroinflammatory Biomarkers for Traumatic Brain Injury Diagnosis and Prognosis: A TRACK-TBI Pilot Study

**DOI:** 10.1089/neur.2022.0060

**Published:** 2023-03-24

**Authors:** John K. Yue, Firas H. Kobeissy, Sonia Jain, Xiaoying Sun, Ryan R.L. Phelps, Frederick K. Korley, Raquel C. Gardner, Adam R. Ferguson, J. Russell Huie, Andrea L.C. Schneider, Zhihui Yang, Haiyan Xu, Cillian E. Lynch, Hansen Deng, Miri Rabinowitz, Mary J. Vassar, Sabrina R. Taylor, Pratik Mukherjee, Esther L. Yuh, Amy J. Markowitz, Ava M. Puccio, David O. Okonkwo, Ramon Diaz-Arrastia, Geoffrey T. Manley, Kevin K.W. Wang

**Affiliations:** ^1^ Department of Neurosurgery, University of California, San Francisco, San Francisco, California, USA.; ^2^ Brain and Spinal Injury Center, Zuckerberg San Francisco General Hospital, San Francisco, California, USA.; ^3^ Departments of Emergency Medicine, Psychiatry, Neuroscience, and Chemistry, University of Florida, Gainesville, Florida, USA.; ^4^ McKnight Brain Institute, University of Florida, Gainesville, Florida, USA.; ^5^ Center for Neurotrauma, Multiomics and Biomarkers, Morehouse School of Medicine, Atlanta, Georgia, USA.; ^6^ Division of Biostatistics and Bioinformatics, Departments of Family Medicine and Public Health, University of California, San Diego, San Diego, California, USA.; ^7^ Department of Emergency Medicine, University of Michigan, Ann Arbor, Michigan, USA.; ^8^ Department of Neurology, University of California, San Francisco, San Francisco, California, USA.; ^9^ Department of Neurology, Epidemiology, and Informatics, University of Pennsylvania Perelman School of Medicine, Philadelphia, Pennsylvania, USA.; ^10^ Department of Biostatistics, Epidemiology, and Informatics, University of Pennsylvania Perelman School of Medicine, Philadelphia, Pennsylvania, USA.; ^11^ Department of Neurosurgery, University of Pittsburgh Medical Center, Pittsburgh, Pennsylvania, USA.; ^12^ Department of Radiology and Biomedical Imaging, University of California, San Francisco, San Francisco, California, USA.

**Keywords:** acute phase reactant, alarmin, cytokine, neuroinflammation, prognosis, traumatic brain injury

## Abstract

The relationship between systemic inflammation and secondary injury in traumatic brain injury (TBI) is complex. We investigated associations between inflammatory markers and clinical confirmation of TBI diagnosis and prognosis. The prospective TRACK-TBI Pilot (Transforming Research and Clinical Knowledge in Traumatic Brain Injury Pilot) study enrolled TBI patients triaged to head computed tomography (CT) and received blood draw within 24 h of injury. Healthy controls (HCs) and orthopedic controls (OCs) were included. Thirty-one inflammatory markers were analyzed from plasma. Area under the receiver operating characteristic curve (AUC) was used to evaluate discriminatory ability. AUC >0.7 was considered acceptable. Criteria included: TBI diagnosis (vs. OC/HC); moderate/severe vs. mild TBI (Glasgow Coma Scale; GCS); radiographic TBI (CT positive vs. CT negative); 3- and 6-month Glasgow Outcome Scale-Extended (GOSE) dichotomized to death/greater relative disability versus less relative disability (GOSE 1–4/5–8); and incomplete versus full recovery (GOSE <8/ = 8). One-hundred sixty TBI subjects, 28 OCs, and 18 HCs were included. Markers discriminating TBI/OC: HMGB-1 (AUC = 0.835), IL-1b (0.795), IL-16 (0.784), IL-7 (0.742), and TARC (0.731). Markers discriminating GCS 3–12/13–15: IL-6 (AUC = 0.747), CRP (0.726), IL-15 (0.720), and SAA (0.716). Markers discriminating CT positive/CT negative: SAA (AUC = 0.767), IL-6 (0.757), CRP (0.733), and IL-15 (0.724). At 3 months, IL-15 (AUC = 0.738) and IL-2 (0.705) discriminated GOSE 5–8/1–4. At 6 months, IL-15 discriminated GOSE 1–4/5–8 (AUC = 0.704) and GOSE <8/ = 8 (0.711); SAA discriminated GOSE 1–4/5–8 (0.704). We identified a profile of acute circulating inflammatory proteins with potential relevance for TBI diagnosis, severity differentiation, and prognosis. IL-15 and serum amyloid A are priority markers with acceptable discrimination across multiple diagnostic and outcome categories. Validation in larger prospective cohorts is needed. ClinicalTrials.gov Registration: NCT01565551

## Introduction

Traumatic brain injury (TBI) affects an estimated 2 to 5 million people annually in the United States and 70 million worldwide.^1–3^ A significant subpopulation suffers persistent deficits, leading to loss of livelihood and societal costs.^4–6^ Determining the extent of acute injury and long-term prognosis remains challenging because of heterogeneity in patient characteristics, pathoanatomical subtypes, and local or systemic inflammatory responses that drive secondary injury. Objective, reliable, and efficient tools for TBI diagnosis, triage, and prognosis are greatly needed.

A major milestone was reached in 2018 when the U.S. Food and Drug Administration cleared two central nervous system (CNS)-specific biomarkers, glial fibrillary acidic protein (GFAP) and ubiquitin C-terminal hydrolase (UCH-L1), to aid in TBI evaluation.^7^ Literature on biomarker-assisted TBI evaluation, before and after the approval of GFAP and UCH-L1, has focused on brain-enriched molecules, which have good discrimination for TBI severity.^8^ However, because systemic inflammation can cause secondary brain injury,^9^ it is also important to identify promising non-CNS-specific biomarkers in TBI diagnosis and prognosis.

Systemic biomarkers have potential utility in detecting not only the presence of brain injury, but also its evolution from acute to subacute and chronic phases. Primary TBI triggers reactive astrogliosis, recruitment of local and systemic immune cells to damaged neural tissue, and release of pro- and anti-inflammatory cytokines that mediate cellular repair, secondary injury, and neurodegeneration.^10,11^ TBI induces and modulates circulating levels of selected cytokines, chemokines, and alarmins that activate secondary injury cascades and cause blood–brain barrier (BBB) breakdown, cytotoxic and vasogenic edema, excessive immune cell infiltration, and neuronal apoptosis.^12^ Collectively, certain cytokines—small proteins that modulate cell-cell communication and immune reactions (e.g., interleukins [ILs], tumor necrosis factors [TNF]), chemokines—a subclass of cytokines that recruits immune cells toward lesions (e.g., macrophage-associated proteins), and alarmins—damage-associated molecular patterns that trigger and amplify inflammatory cascades (“danger signals”),^13,14^ constitute key signaling molecules that bridge primary and secondary TBI, with potentially dynamic roles in TBI outcome.

One recent example of an alarmin with promise in TBI detection, progression, and outcome is high mobility group box 1 (HMGB-1). HMGB-1 is a ubiquitous nuclear protein released by damaged cells that initiates host defenses in acute tissue/organ damage and has been found to be prognostic of the degree of residual function in injured tissue.^15^ Circulating HMGB-1 activates liver-derived acute phase reactants, such as serum amyloid A (SAA) and C-reactive protein (CRP), which in turn propagate multiple cytokine and chemokine cascades to amplify systemic and neuroinflammation.^16^ Activation of specific secondary injury cascades may preferentially affect long-term outcome after TBI, as evidenced by the association observed between higher HMGB-1 and poorer 6-month Glasgow Outcome Scale in pediatric TBI,^17^ underscoring the potential value of neuroinflammatory markers as therapeutic targets in TBI recovery.^18^ Indeed, neuroinflammation may explain why some TBI patients develop persistent deficits whereas others progress to good recovery.

Recent research has targeted the blockade of TBI-specific cytokines, using receptor antagonists and monoclonal antibodies to dampen overactive inflammatory responses and facilitate neuroprotection after CNS trauma.^19,20^ Determining the precise cellular interactions among candidate cytokines, chemokines, and alarmins during acute TBI will aid in discovering the inflammatory endophenotypes relevant to TBI diagnosis and outcome, similar to recent successes in traumatic microvascular and neurodegenerative studies.^21,22^

Identification of promising neuroinflammatory markers is the critical next step for determining therapeutic targets in cellular injury pathways after TBI. Using a multi-marker panel with robust and reliable assays from pre-clinical and clinical data,^23–25^ we aimed to identify acute inflammatory markers (cytokines, chemokines, and alarmins) suitable for next-phase validation in TBI detection and outcome, in a prospective cohort of acute TBI subjects and controls.

## Methods

### Study overview and informed consent

The prospective, multi-center TRACK-TBI Pilot (Transforming Research and Clinical Knowledge in Traumatic Brain Injury Pilot) study enrolled patients with external force trauma to the head who presented to one of three participating U.S. level 1 trauma centers and received a clinically indicated head computed tomography (CT) scan within 24 h of injury between years 2010 and 2012, as previously described (ClinicalTrials.gov Registration: NCT01565551).^26^ TRACK-TBI Pilot applied the American College of Emergency Physicians/Centers for Disease Control and Prevention guidelines for obtaining head CTs,^27^ and data were collected using the National Institutes of Health (NIH) TBI Common Data Elements (CDEs), version 1.^28^ Exclusion criteria were pregnancy, ongoing life-threatening disease (e.g., end-stage malignancy), police custody, involuntary psychiatric hold, and non-English speakers.^28^ A subset of TRACK-TBI Pilot subjects underwent venous blood draw within 24 h of injury and 3- and 6-month outcomes by structured interview.

Eligible subjects were enrolled by convenience sampling at each participating site. Institutional review board (IRB) approval was obtained at each site, and the overall study received approval from the IRB of record at the University of California, San Francisco (UCSF; Protocol No.: 10-00111).^28^ Informed consent was obtained before enrollment. For subjects unable to provide consent because of the severity of their injury, consent was obtained from their legally authorized representative or surrogate next of kin. Subjects were reconsented, if cognitively able, during their clinical care and/or follow-up time points regarding continuation in study participation.^28^

### Study subjects and blood sample processing

The current analysis included a subset of TRACK-TBI Pilot subjects who underwent blood draw within 24 h of injury and had unused samples available for analysis. Blood collection and processing in TRACK-TBI Pilot were performed in accordance with the NIH TBI CDEs, as previously described.^28,29^ Four to 8 mL of whole blood was collected by peripheral venipuncture using dipotassium ethylene diamine tetraacetic acid vacutainer tubes (Becton, Dickinson and Company, Franklin Lakes, New Jersey, U.S.), which are the standard blood collection tubes used for clinical care at our institution. Fresh blood samples were placed on ice for 5 min, then processed by centrifuge at 4000 revolutions per minute for 7 min. Plasma was aliquoted into multiple 250-μL cryovials per patient and stored in −80°C freezers at the UCSF DNA Bank (San Francisco, CA). The process from blood draw to storage at −80°C was completed within 1 h. Plasma samples were stored until they were retrieved for assay analysis; the plasma samples used in the current analysis received one freeze-thaw cycle over their lifetime.

In addition, orthopedic injury controls (OCs) and healthy controls (HCs) were recruited by convenience sampling and patient availability. OCs were patients who presented to a participating trauma center within 24 h of acute trauma to their limbs, pelvis, and/or thorax and had an Abbreviated Injury Scale score <4 for those regions. OCs did not have loss or alteration of consciousness, peritraumatic amnesia, or other clinical findings suggestive of TBI and did not undergo a head CT as part of their clinical care. OCs underwent the same informed consent procedure as TBI patients and received a venous blood draw within 24 h of injury. HCs without acute injuries were recruited from the community through an existing relationship with a TRACK-TBI participant or approved public advertisement within TRACK-TBI institutions and received a venous blood draw after informed consent was obtained. HCs were excluded if they had a self-reported history of TBI or polytrauma within 12 months of enrollment. Blood collection and processing for OCs and HCs were identical to TBI patients.

### Plasma biomarker analyses

We assembled a multi-marker panel of 31 priority inflammatory markers for investigation. Plasma was extracted from blood samples as previously described.^30^ All biomarker assays were run in a blinded fashion at the University of Florida Biomarker Laboratory supervised by the senior author K.K.W.W. (Gainesville, FL). Thirty inflammatory markers were analyzed using pre-made Meso Scale Discovery (MSD) V-Plex Panels: Proinflammatory Panel 1 (Catalog #K15049D-1), Cytokine Panel 1 (#K15050D-1), Chemokine Panel 1 (#K15047D-1), and Vascular Injury Panel 2 (#K15198D-1) without using its vascular cell adhesion molecule (VCAM) assay (Meso Scale Diagnostics, LLC, Rockville, MD).^23^ Though the MSD Vascular Injury Panel 2 and other V-Plex Panels often included vascular and angiogenesis markers, such as VCAM, types of vascular endothelial growth factors, fibroblast growth factor, and others, these were not included in the current analysis because of being out of scope.

We report data on the following markers: CRP, eotaxin, eotaxin-3, interferon gamma-induced protein 10 (IP-10), interferon-γ (IFN-γ), intercellular adhesion molecule 1 (ICAM-1), IL-1a, IL-1b, IL-2, IL-4, IL-5, IL-6, IL-7, IL-8, IL-10, IL-12/IL-23 p40 protein (IL-12/IL-23p40), IL-12 p70 protein (IL-12p70), IL-13, IL-15, IL-16, IL-17a, macrophage-derived chemokine (MDC), macrophage inflammatory protein 1a (MIP-1a), MIP-1b, monocyte chemoattractant protein-1 (MCP-1), MCP-4, SAA, thymus- and activation-regulated chemokine (TARC), TNF-a, and TNF-b. MSD does not provide an assay for HMGB-1, and we selected the Shino-Test HMGB-1 enzyme-linked immunosorbent assay as a reliable assay because of its wide usage in clinical medicine studies (catalog no.: ST51011; Shino-Test Corporation, Japan, available through Tecan, Incorporated, Morrisville, NC).^15,31,32^

Biomarkers were run in duplicate according to manufacturing instructions, and the average value of the duplicates was used as the final value for each biomarker. The intra- and interassay coefficients of variation are provided in Supplementary Table S1. The lower limit of detection (LLOD) and dynamic range for each MSD biomarker are available at the MSD website^33^ and are reported in Supplementary Table S1. The HMGB-1 assay has a dynamic range of 0.31–160 ng/mL and an LLOD of 0.15 ng/mL. Values below LLOD were not used in the final data analysis. Biomarker concentrations are reported in pg/mL, with the exception of HMGB-1, which is reported in ng/mL.

### Statistical analysis

Biomarker levels were summarized and compared by diagnostic groups. Comparisons were made using the Wilcoxon rank-sum test because of the skewness of the biomarkers' distribution and relatively small sample sizes. The pair-wise Spearman correlation was calculated and plotted between biomarkers among the TBI cases. Median and first to third quartile (Q1–Q3) were reported for descriptive variables, unless otherwise specified. Receiver operating characteristic (ROC) analyses were conducted to assess the performance of each biomarker in discriminating TBI versus OC, TBI versus HC, GCS 3–12 versus GCS 13–15, and CT positive (CT^+^) versus CT negative (CT^–^). ROC analyses were also performed to evaluate the ability of each biomarker to predict 3- and 6-month outcome assessed by the Glasgow Outcome Scale-Extended (GOSE), which consists of an ordinal score from 1 to 8 without units (1 = dead, 2 = vegetative state, 3 = lower severe disability, 4 = upper severe disability, 5 = lower moderate disability, 6 = upper moderate disability, 7 = lower good recovery, and 8 = upper good recovery) and is widely used as the standard measure for functional outcome after TBI.^34,35^ Outcome was dichotomized in two ways: 1) death/greater relative disability (GOSE 1–4: death or severe disability) versus less relative disability (GOSE 5–8: moderate disability or good recovery) and 2) incomplete recovery (GOSE <8) versus full recovery (GOSE = 8), as shown in earlier studies.^36,37^

Area under the ROC curve (AUC) was calculated with 95% confidence intervals. In general, an AUC of 0.5 suggests no discrimination, 0.7–0.8 is considered acceptable, 0.8–0.9 is considered good, and >0.9 is considered excellent.^38^ We adopted an AUC threshold of >0.7 to identify candidate markers with acceptable discrimination for TBI diagnosis and prognosis. Because this was an exploratory secondary analysis of existing data, with known limitations in sample size of TBI patients, OCs, and HCs, *a priori* and *post hoc* power calculations were not performed. Statistical significance was assessed at *p* < 0.05. Analyses were performed using R version 4.1.2.

## Results

### Demographic and clinical data

The analytical cohort included 160 subjects with TBI, 28 OCs, and 18 HCs. Mean age was 44.2 years, and 65% (104 of 160) were male. Seventy-nine percent (124 of 160) presented with GCS 13–15, and 49.4% of patients (79 of 160) had intracranial injuries on initial head CT. At 3 months, 80% (128 of 160) of subjects completed the GOSE; median was 7 (Q1–Q3: 5–8), 18% (23 of 128) had death/greater relative disability (GOSE 1–4), and 25.8% (33 of 128) had full recovery (GOSE = 8). At 6 months, 74.3% (119 of 160) of patients completed the GOSE; median was 7 (Q1–Q3: 5–7), 15.1% (18 of 119) had death/greater relative disability, and 24.4% (29 of 119) had full recovery. Full demographic and clinical data are presented in Table 1.

**Table 1.** Demographic and Clinical Characteristics of TBI Subjects

Variable	% (of* N* = 160)
Age (years)	
Mean (SD)	44.2 (18.0)
Sex	
Male	104 (65.0%)
Female	56 (35.0%)
Race	
White/Caucasian	131 (81.9%)
African-American/African	12 (7.5%)
Other race	17 (10.6%)
Ethnicity	
Hispanic	26 (16.6%)
Non-Hispanic	131 (83.4%)
Education	
Below high school	18 (11.8%)
High school graduate	93 (61.2%)
College degree or above	41 (27.0%)
Employment	
Full time	59 (38.6%)
Part time	20 (13.1%)
Unemployed	38 (24.8%)
Retired/student/disabled	36 (23.5%)
Loss of consciousness	
No	42 (26.6%)
Yes	101 (63.9%)
Unknown	15 (9.5%)
Post-traumatic amnesia	
No	49 (31.0%)
Yes	86 (54.4%)
Unknown	23 (14.6%)

Initial GCS	% (of* N* = 157)
3–12	33 (21.0%)
13–15	124 (79.0%)
Intracranial injury on CT	
Absent (CT negative)	81 (50.6%)
Present (CT positive)	79 (49.4%)

3-month outcome (GOSE)	% (of* N* = 128)
Median (Q1, Q3)	7 (5, 8)
Death/greater relative disability (GOSE 1–4)	23 (18.0%)
Less relative disability (GOSE 5–8)	105 (82.0%)
Incomplete recovery (GOSE <8)	95 (74.2%)
Full recovery (GOSE = 8)	33 (25.8%)

6-month outcome (GOSE)	% (of* N* = 119)
Median (Q1, Q3)	7 (5, 7)
Death/greater relative disability (GOSE 1–4)	18 (15.1%)
Less relative disability (GOSE 5–8)	101 (84.9%)
Incomplete recovery (GOSE <8)	90 (75.6%)
Full recovery (GOSE = 8)	29 (24.4%)

Proportions are displayed for demographic and clinical characteristics of 160 acute TBI subjects with neuroinflammatory biomarker data. Initial GCS and 3- and 6-month outcome were obtained in a subset of patients with their corresponding sample sizes shown. Three- and 6-month GOSE are reported as their ordinal score.

CT, computed tomography; GCS, Glasgow Coma Scale; GOSE, Glasgow Outcome Scale-Extended; Q1, first quartile; Q3, third quartile; SD, standard deviation; TBI, traumatic brain injury.

### Clinical diagnosis, traumatic brain injury severity, and radiographic diagnosis

Acute inflammatory biomarkers with acceptable discriminatory ability (AUC >0.7) for clinical diagnosis of TBI, TBI severity, and radiographic TBI are described below and in detail in Table 2.

**Table 2.** Markers Discriminating TBI Clinical Diagnosis and Severity

Clinical diagnosis: TBI vs. HC				
Biomarker	AUC	TBI	HC	Sig. (*p*)
IL-6	0.924 [0.880–0.967]	1.47 [0.55–4.07] pg/mL	0.15 [0.10–0.22] pg/mL	<0.001
IL-10	0.863 [0.804–0.922]	0.17 [0.10–0.39] pg/mL	0.05 [0.04–0.08] pg/mL	<0.001
HMGB-1	0.860 [0.802–0.919]	47.48 [24.35–146.79] ng/mL	20.77 [14.88–20.77] ng/mL	<0.001
IL-4	0.819 [0.731–0.907]	0.09 [0.07–0.15] pg/mL	0.06 [0.06–0.07] pg/mL	<0.001
IL-7	0.764 [0.637–0.891]	0.61 [0.25–1.29] pg/mL	2.32 [0.90–3.67] pg/mL	<0.001
IL-8	0.764 [0.666–0.862]	3.46 [1.53–12.58] pg/mL	1.29 [0.50–1.64] pg/mL	0.001
TARC	0.749 [0.626–0.872]	16.23 [10.49–29.74] pg/mL	40.63 [22.08–56.31] pg/mL	<0.001
IL-5	0.748 [0.621–0.874]	0.37 [0.26–0.49] pg/mL	0.24 [0.16–0.35] pg/mL	<0.001
IL-16	0.727 [0.642–0.813]	146.17 [107.02–309.52] pg/mL	110.04 [98.74–114.16] pg/mL	0.002

Clinical diagnosis: TBI vs. OC				
Biomarker	AUC	TBI	OC	Sig. (*p*)
HMGB-1	0.835 [0.774–0.895]	47.48 [24.35–146.79] ng/mL	20.77 [16.05–22.59] ng/mL	<0.001
IL-1b	0.795 [0.729–0.860]	0.09 [0.03–0.46] pg/mL	0.03 [0.02–0.03] pg/mL	<0.001
IL-16	0.784 [0.709–0.858]	146.17 [107.02–309.52] pg/mL	98.52 [91.40–115.00] pg/mL	<0.001
IL-7	0.742 [0.651–0.833]	0.61 [0.25–1.29] pg/mL	1.62 [1.14–2.04] pg/mL	<0.001
TARC	0.731 [0.637–0.825]	16.23 [10.49–29.74] pg/mL	27.32 [21.61–52.27] pg/mL	<0.001

Clinical severity: GCS 3–12 vs. 13–15				
Biomarker	AUC	GCS 3–12	GCS 13–15	Sig. (*p*)
IL-6	0.747 [0.650–0.844]	4.91 [1.94–10.29] pg/mL	1.12 [0.45–2.51] pg/mL	<0.001
CRP	0.726 [0.618–0.834]	14,329.96 [347.48–43,444.93] pg/mL	394.99 [219.00–4014.48] pg/mL	<0.001
IL-15	0.720 [0.607–0.833]	1.11 [0.61–1.44] pg/mL	0.57 [0.41–0.86] pg/mL	<0.001
SAA	0.716 [0.608–0.825]	123,228.06 [8338.68–141,280.09] pg/mL	2842.89 [1912.89–18,437.41] pg/mL	<0.001

Radiographic severity: CT^+^ vs. CT^–^				
Biomarker	AUC	CT^+^	CT^–^	Sig. (*p*)
SAA	0.767 [0.693–0.840]	71,255.85 [2380.56–140,531.01] pg/mL	2233.36 [1813.87–8010.34] pg/mL	<0.001
IL-6	0.757 [0.682–0.833]	2.48 [1.24–8.68] pg/mL	0.66 [0.37–2.00] pg/mL	<0.001
CRP	0.733 [0.652–0.813]	7265.86 [297.77–33,780.47] pg/mL	265.30 [214.91–829.27] pg/mL	<0.001
IL-15	0.724 [0.644–0.804]	0.89 [0.52–1.24] pg/mL	0.51 [0.37–0.71] pg/mL	<0.001

AUCs reflect the ability of each biomarker to discriminate between respective categories of clinical and radiographic diagnosis for TBI. Markers with AUC >0.7 (threshold for acceptable discriminatory ability) and their respective 95% confidence intervals are shown for each category in column 2. Median and Q1–Q3 values (in ng/mL or pg/mL) for each biomarker are shown in columns 3 and 4.

AUC, area under the receiver-operating characteristic curve; CRP, C-reactive protein; CT, computed tomography; GCS, Glasgow Coma Scale; HC, healthy control; HMGB-1, high mobility group box 1; IL, interleukin; OC, orthopedic control; Q1, first quartile; Q3, third quartile; SAA, serum amyloid protein A; TARC, thymus- and activation-regulated chemokine; TBI, traumatic brain injury.

Biomarkers with acceptable discrimination between TBI versus HC, with higher values in TBI, included: IL-6 (AUC = 0.924), IL-10 (0.863), HMGB-1 (0.860), IL-4 (0.819), IL-8 (0.764), IL-5 (0.748), and IL-16 (0.727). Biomarkers with acceptable discrimination between TBI versus HC, with lower values in TBI, included: IL-7 (0.764) and TARC (0.749).

Biomarkers with acceptable discrimination between TBI versus OC, with higher values in TBI, included: HMGB-1 (AUC = 0.835), IL-1b (0.795), and IL-16 (0.784). Biomarkers with acceptable discrimination between TBI versus OC, with lower values in TBI, included: IL-7 (0.742) and TARC (0.731).

Biomarkers with acceptable discrimination between moderate-to-severe versus mild TBI included: IL-6 (AUC = 0.747), CRP (0.726), IL-15 (0.720), and SAA (0.716). Of these, all markers were higher in the moderate-to-severe TBI.

Biomarkers with acceptable discrimination for radiographic TBI included: SAA (AUC = 0.767), IL-6 (0.757), CRP (0.733), and IL-15 (0.724). Of these, all markers were higher in CT-positive patients.

### 3- and 6-month prognosis/outcome

Inflammatory biomarkers with acceptable discriminatory ability for 3- and 6-month outcome are described below and in Table 3.

**Table 3.** Predictors of 3- and 6-Month Outcome Post-TBI

3-month death/greater relative disability vs. less relative disability (GOSE 1–4 vs. 5–8)				
Biomarker	AUC	GOSE 1–4 (*N* = 23)	GOSE 5–8 (*N* = 105)	Sig. (*p*)
IL-15	0.738 [0.615–0.861]	1.11 [0.69–1.43] pg/mL	0.55 [0.39–0.87] pg/mL	<0.001
IL-2	0.705 [0.587–0.823]	0.10 [0.08–0.17] pg/mL	0.08 [0.07–0.10] pg/mL	0.002

6-month death/greater relative disability vs. less relative disability (GOSE 1–4 vs. 5–8)				
Biomarker	AUC	GOSE 1–4 (*N* = 18)	GOSE 5–8 (*N* = 101)	Sig. (*p*)
IL-15	0.704 [0.557–0.850]	1.11 [0.62–1.39] pg/mL	0.56 [0.40–3.71] pg/mL	0.006
SAA	0.704 [0.564–0.843]	90,752.53 [10,228.08–142,520.26] pg/mL	3001.77 [1917.35–66,046.15] pg/mL	0.006

6-month incomplete vs. full recovery (GOSE <8 vs. = 8)				
Biomarker	AUC	GOSE <8 (*N* = 90)	GOSE = 8 (*N* = 29)	Sig. (*p*)
IL-15	0.711 [0.607–0.815]	0.69 [0.48–1.20] pg/mL	0.43 [0.30–0.65] pg/mL	<0.001

AUCs reflect the ability of each biomarker to discriminate between respective categories of 3- and 6-month outcome after TBI. Markers with AUC >0.7 (threshold for acceptable discriminatory ability) and their respective 95% confidence intervals are shown for each category in column 2. Median and Q1–Q3 values (in pg/mL) for each biomarker are shown in columns 3 and 4. No biomarker had a discriminatory ability above threshold for 3-month incomplete vs. full recovery.

AUC, area under the receiver-operating characteristic curve; GOSE, Glasgow Outcome Scale-Extended; IL, interleukin; Q1, first quartile; Q3, third quartile; SAA, serum amyloid protein A; TBI, traumatic brain injury.

For 3-month death/greater relative disability (GOSE 1–4) versus less relative disability (GOSE 5–8), biomarkers with acceptable discrimination included: IL-15 (AUC = 0.738) and IL-2 (0.705). Biomarker values were higher in those with death/greater relative disability. No biomarker had discriminatory ability above threshold for 3-month incomplete versus full recovery (GOSE <8 vs. GOSE = 8).

For 6-month death/greater relative disability versus less relative disability, biomarkers with acceptable discrimination included: IL-15 (AUC = 0.704) and SAA (0.704). Biomarker values were higher in patients with death/greater relative disability. For 6-month incomplete versus full recovery, the only biomarker with acceptable discrimination was IL-15 (AUC = 0.711), and biomarker values were higher in those with incomplete recovery.

Complete data with AUCs for all 31 biomarkers across clinical and diagnostic categories, and 3- and 6-month outcome categories, are provided in Supplementary Table S2.

### Correlations between biomarkers

**FIG. 1.**
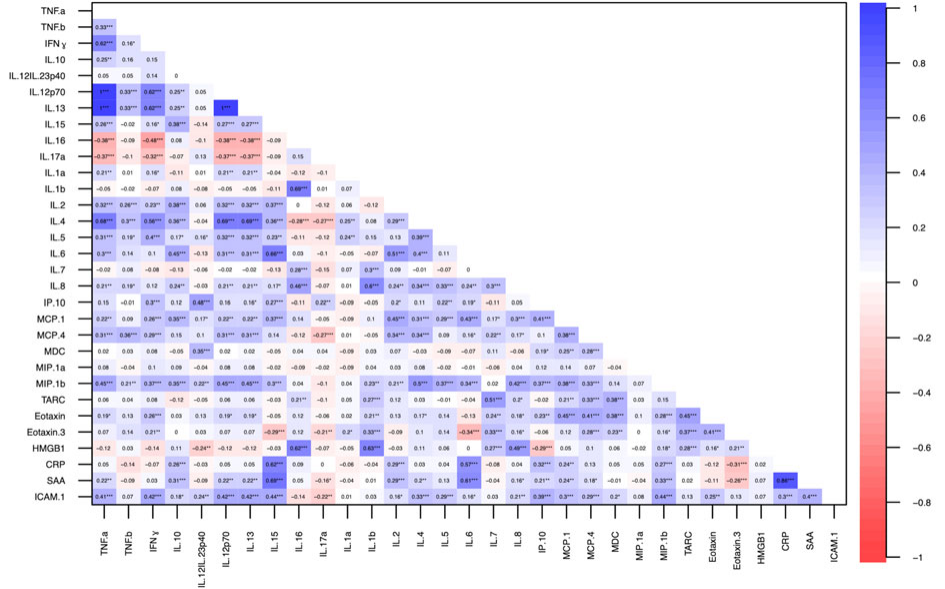
Correlation matrix for 31 neuroinflammatory biomarkers after acute TBI. Spearman's correlation matrix is shown for the 31 biomarkers included in the current study. Correlation ranges from −1 to +1. Spearman's correlation was considered “moderate” in the 0.6–0.8 range and “strong” if >0.8. CRP, C-reactive protein; HMGB-1, biomarker high mobility group box 1; ICAM-1, intercellular adhesion molecule 1; IFN-γ, interferon-γ; IL, interleukin; IL-12/IL-23p40, IL-12/IL-23 p40 protein; IL-12 p70, IL-12 p70 protein; IP-10, interferon gamma-induced protein 10; MCP, monocyte chemoattractant protein; MDC, macrophage-derived chemokine; MIP-1a, macrophage inflammatory protein 1a; SAA, serum amyloid A; TARC, thymus- and activation-regulated chemokine; TBI, traumatic brain injury; TNF, tumor necrosis factor.

Spearman's correlation matrix was used to evaluate potential collinearity (redundancy) among diagnostic and prognostic markers (Fig. 1). Among markers within the same category of diagnostic or prognostic discrimination (in Tables 2 and 3), several correlations were of moderate strength (0.60–0.79), including IL-15/SAA (0.69), HMGB-1/IL-1b (0.63), HMGB-1/IL-16 (0.62), IL-15/CRP (0.62), and SAA/IL-6 (0.61). The SAA/CRP correlation (0.86) was the only one to exceed moderate strength.

## Discussion

TBI patients show upregulated neuroinflammatory genes and increased expression of cytokines, chemokines, the alarmin HMGB-1, and acute phase reactants (SAA, CRP).^39,40^ We identified a distinct profile of neuroinflammatory proteins detectable in the systemic circulation within 24 h of acute TBI, with potential utility for objective TBI detection, severity differentiation, and prognosis. Identification of markers able to discriminate both clinical/radiographic TBI severity *and* better/worse outcome is an important step toward the determination of an inflammatory endophenotype in TBI and potential targets for therapeutic modulation.

General TBI diagnostic criteria include external force trauma to the head causing an alteration of consciousness.^41^ TBI severity has been historically defined as “mild, moderate, or severe” based on GCS and head CT results. Whereas “severe” GCS and greater extent of intracranial injury portend a worse prognosis, their sensitivity for outcome prediction is limited. Objective, quantifiable biomarkers with the ability to determine TBI presence and severity have a wide range of applications, including early detection in pre-hospital settings or where neuroimaging is unavailable, confirmation of injury (e.g., patient with equivocal CT and persistent neurological deficit), and triage to appropriate resources ranging from observation to intensive care unit admission. Though CNS-specific biomarkers such as GFAP and UCH-L1 have been qualified for the evaluation of TBI,^42,43^ neuroinflammatory biomarkers have the added importance of comprising distinct biochemical and molecular pathways that contribute to secondary injury cascades that cross into subacute and chronic phases, and become a continuum with recovery and outcome. Validation and qualification of robust neuroinflammatory markers can enable the development of a high-yield TBI biomarker panel to serve as primary or adjunct tools for diagnosis. Downstream inflammatory cascades not only contribute to outcome prediction, but may also be promising targets for therapeutic modulation in clinical trials.

In our study, few markers showed acceptable concurrent discriminability for both TBI diagnosis and prognosis. One marker was IL-15, which showed acceptable discriminability (AUC >0.7) across TBI severity, radiographic injury, and 3- and 6-month GOSE 1–4 versus 5–8. IL-15 is a proinflammatory cytokine expressed centrally by neuronal and glial cells, peripherally in macrophages and monocytes, and exists in both intracellular and secretory forms.^44^ Although it has low BBB permeability, peripheral IL-15 activates multiple CNS signaling pathways.^45^ IL-15 is robustly upregulated in neuroinflammation, induces reactive gliosis, and modulates gamma-amino butyric acid and serotonin transmission, affecting mood, memory, sleep, and activity. These cascades are relevant to acute inflammation and as contributors to persistent cognitive, behavioral, and functional disability. Substantial progress has been made in the IL-15 blockade in cellular and animal models of various neuroinflammatory conditions.^46,47^ If IL-15 is causally linked to secondary neurological injury in TBI, IL-15 may be a candidate for neuroprotective blockade in human trials.

SAA is the second marker with acute and long-term implications (AUC >0.7 for clinical and radiographic TBI severity, as well as 6-month GOSE 1–4 vs. 5–8). As with IL-15, SAA may represent another link between acute injury and long-term inflammatory cascades. SAA is released into the circulation after major injury or infection, induces monocyte and neutrophil migration, and stimulates the production and release of cytokines, chemokines, and matrix metalloproteinases.^48,49^ These all have broad downstream effects in the activation of transcription factors and epigenetic regulation not only in proinflammatory states, but also for subsequent homeostasis during inflammation.^48,49^ Murine models have demonstrated that SAA levels correspond to injury severity after controlled cortical impact, with important roles in microglial recruitment and neutrophil infiltration that lead to substantial secondary injury.^50^ In our data, the concentration of SAA was 43-fold higher in GCS 3–12 versus GCS 13–15, and 30-fold higher in CT^+^ vs. CT^–^ patients, congruent with its role as an acute phase reactant. SAA has been shown to transiently increase up to 1000-fold during acute injury, although it should return to baseline levels after the insult has resolved.^51^ In our study, patients with 6-month GOSE 1–4 had a 30-fold acute elevation of SAA compared with GOSE 5–8, underscoring the potential role of SAA in an inflammatory endophenotype connecting persistent inflammation with poor long-term outcome.

Recent literature in patients with cerebral microvascular disease has implicated increased SAA and CRP with a cluster of proinflammatory cytokines (IL-6, IL-8, IL-10, and TNF-a) in persistent anxiety.^52^ SAA and CRP correlated strongly in our data set, but differed in the discriminability of TBI severity. Though the ubiquitous role of SAA in acute phase response makes it a more challenging therapeutic target, there is the potential for research into the neuroprotective blockade of molecules either up- or downstream to SAA in various pathways.

In contrast to the small subset of markers predictive of outcomes, the markers associated with primary injury are more diverse. The five diagnostic markers of brain-specific trauma (TBI vs. OC: HMGB-1, IL-1b, IL-7, IL-16, and TARC) did not overlap with markers of TBI severity by GCS or CT criteria (SAA, CRP, IL-6, and IL-15), whereas markers for the latter were identical. This suggests that whereas inflammatory signals are induced at the time of injury, distinct clusters of markers may be induced by different TBI severities and/or injury patterns identifiable by CT. This phenomenon is reassuring, given that it suggests that these cytokine levels are not broadly and indiscriminately altered after TBI, but may be divisible into distinct biomarker profiles that are able to differentiate nuanced clinical correlates.

On correlation analysis, analytical “pairs” of inflammatory markers emerged. IL-15 showed moderate correlations with SAA and CRP, implicating its involvement across acute-phase cellular cascades. The alarmin HMGB-1 was associated with IL-1b and IL-16; HMGB-1 increases chemotaxis and activation of leukocytes *ex vivo*, triggers microglial activation and neuroinflammation, and has been closely associated with detrimental effects of brain injury in traumatic and non-traumatic animal and cellular models.^53^ TBI-induced microglial activation and increased expression of proinflammatory mediators, such as HMGB-1 and IL-6, have been associated with cerebral edema and neurological deficits.^16,54^ Our results support the likelihood of HMGB-1 as a marker for brain-specific trauma in humans. The correlations identified in our study underscore the complex crosstalk among markers of neuroinflammation and secondary injury and inform the development of biomarker “panels” for validation in acute and chronic TBI.

Finally, our data showed an overlap between markers for brain-specific trauma (TBI vs. OC) with TBI versus HC. Given the multitude and variability of systemic inflammatory pathways activated by trauma, the identification of neuroinflammatory markers with a discriminatory potential for diagnosis and prognosis should focus on brain-specific, rather than generalized, trauma.

### Limitations

We recognize several limitations. We performed an exploratory secondary analysis of existing data in a relatively small sample of TBI patients, with fewer numbers of OCs and HCs attributable to limitations in convenience sampling and recruitment. Confirmatory studies with larger numbers of TBI patients and controls encompassing diverse demographics and injury severities are needed, with the additional goal of robustly quantifying differences in biomarker levels between TBIs with and without polytrauma. Changes in biomarker levels as part of non-TBI systemic trauma should also be quantified and accounted for in validation studies. To identify associations for near-term validation and clinical implementation, we dichotomized variables for radiographic injury and functional outcome and used a more stringent cutoff of AUC >0.7 to define “acceptable” discrimination and may have selected out markers with lower AUCs that would have increased with larger sample sizes. Because of the small number of markers above our AUC cutoff, we did not perform multi-variate analyses, which would have provided more definitive yield in larger validation data sets.

We were limited by the assays used for this study, which did not include CNS-based biomarkers (e.g. GFAP, UCH-L1). Our study scope focused on acute inflammatory cytokines, chemokines, and alarmins, and we did not include other classes of markers, such as vascular injury and angiogenesis, that may be relevant to TBI injury cascades and outcome^21,22^ and/or interact with neuroinflammatory cascades. At the time of our study design, some neuroimmune cytokine assays were not yet available at MSD (e.g., IL-31),^55^ which may warrant inclusion in future studies. We recognize that systemic inflammatory markers may be elevated in non-TBI acute and chronic inflammatory conditions (e.g. the acute stress response, autoimmune disorders, infection, malignancy, and others).^56–59^

We were limited by the available data from the TRACK-TBI Pilot study, which did not collect comprehensive data on pre-existing inflammatory conditions; it would be important for validation studies to adjust for these important confounders when interpreting inflammatory biomarker values in the context of TBI diagnosis and prognosis. Important next steps include evaluating for more granular associations among cytokine markers, intracranial injury type and location, multi-dimensional outcomes, and changes in their diagnostic/prognostic ability when combined with CNS-specific biomarkers. Evaluation of temporal cascades of inflammatory biomarkers will clarify their relationship with secondary injury and recovery trajectories. Hypothesis-driven studies with appropriate power calculations should be prioritized. Advanced statistical modeling (e.g., dimension reduction) can identify clusters of markers with improved diagnostic or prognostic discriminability and elucidate the underlying “neuroinflammatory endophenotype” that may modulate TBI outcome. These limitations await imminent validation studies utilizing the 18-center prospective TRACK-TBI consortium (https://tracktbi.ucsf.edu/).

## Conclusion

We identified a distinct profile of inflammatory proteins detectable in the systemic circulation within 24 h of acute TBI, which may be significant for TBI diagnosis, severity differentiation, and prognosis. The proinflammatory cytokine IL-15 and the acute phase reactant SAA had acceptable discriminatory ability for clinical and radiographic TBI, as well as for outcome after TBI. Future research is needed to validate these findings in a larger cohort and understand how levels of these biomarkers change over time as injury evolves from acute to subacute and chronic phases. This understanding may yield potential targets for therapeutic intervention.

## Supplementary Material

Supplemental data

Supplemental data

## TRACK-TBI Investigators

Neeraj Badjatia, MD (Department of Neurology, University of Maryland, Baltimore, MD); Brandon Foreman, MD (Department of Neurology, University of Cincinnati, Cincinnati, OH); Shankar Gopinath, MD (Department of Neurosurgery, Baylor College of Medicine, Houston, TX); Ramesh Grandhi, MD, MS (Department of Neurosurgery, University of Utah, Salt Lake City, UT); Ruchira M. Jha, MD, MSc (Department of Neurology, Barrow Neurological Institute, Phoenix, AZ); Hester F. Lingsma, PhD (Department of Public Health, Erasmus Medical Center, Rotterdam, The Netherlands); Christopher Madden, MD (Department of Neurosurgery, University of Texas Southwestern Medical Center, Dallas, TX); Debbie Y. Madhok, MD (Department of Emergency Medicine, University of California San Francisco, San Francisco, CA); Michael A. McCrea, PhD (Department of Neurosurgery, Medical College of Wisconsin, Milwaukee, WI); Randall Merchant, PhD (Department of Anatomy and Neurobiology, Virginia Commonwealth University, Richmond, VA); Lindsay D. Nelson, PhD (Department of Neurosurgery, Medical College of Wisconsin, Milwaukee, WI); Laura B. Ngwenya, MD, PhD (Department of Neurosurgery, University of Cincinnati, Cincinnati, OH); Claudia S. Robertson, MD (Department of Neurosurgery, Baylor College of Medicine, Houston, TX); Richard B. Rodgers, MD (Goodman Campbell Brain and Spine, Indianapolis, IN); Gabriela G. Satris, RN, MSN, MSc (Department of Neurosurgery, University of California San Francisco, San Francisco, CA); David M. Schnyer, PhD (Department of Psychology, University of Texas at Austin, Austin, TX); Alex B. Valadka, MD **(**Department of Neurosurgery, University of Texas Southwestern Medical Center, Dallas, TX); Thomas A. van Essen, MD, PhD (Department of Neurosurgery, Leiden University Medical Center, Leiden, The Netherlands); Ross Zafonte, DO (Department of Rehabilitation Medicine, Harvard Medical School, Boston, MA).

## Abbreviations Used

AUC: area under the receiver-operating characteristic curve
BBB: blood–brain barrier
CDes: Common Data Elements
CNS: central nervous system
CRP: C-reactive protein
CT: computed tomography
GCS: Glasgow Coma Scale
GFAP: glial fibrillary acidic protein
GOSE: Glasgow Outcome Scale Extended;
HC: healthy control
HMGB-1: high mobility group box 1
ICAM-1: intercellular adhesion molecule 1
IFN-γ: interferon-γ
IL: interleukin
IP-10: interferon gamma-induced protein 10
IRB: institutional review board
LLOD: lower limit of detection
MCP: monocyte chemoattractant protein
MDC: macrophage-derived chemokine
MIP: macrophage inflammatory protein
NIH: National Institutes of Health
OC: orthopedic control
ROC: receiver operating characteristic
SAA: serum amyloid A
TARC: thymus- and activation-regulated chemokine
TBI: traumatic brain injury
TNF: tumor necrosis factor
UCH-L1: ubiquitin C-terminal hydrolase L1
UCSF: University of California, San Francisco
VCAM: vascular cell adhesion molecule
