# Supplementary material for: Neuroinflammatory Biomarkers for Traumatic Brain Injury Diagnosis and Prognosis: A TRACK-TBI Pilot Study
Source: Neurotrauma Rep. 2023 Mar 24;4(1):171–83. doi: 10.1089/neur.2022.0060 (PMC10039275; doi:10.1089/neur.2022.0060)
Supplement: Supplemental data [file Suppl_TableS1.docx]

**Supplemental Table 1. Assay Characteristics, Sensitivity, and Precision**

| **Biomarker** | **Intra-assay %CV** | **Inter-assay %CV** | **LLOD** | **Dynamic Range** |
| --- | --- | --- | --- | --- |
| CRP | 7.5 | 6.9 | 1.33 pg/mL | 1.33 - 49600 pg/mL |
| Eotaxin | 8.6 | 9.7 | 3.26 pg/mL | 3.26 - 1120 pg/mL |
| Eotaxin-3 | 1.7 | 5.7 | 1.77 pg/mL | 1.77 - 3750 pg/mL |
| HMGB-1 | 6.7 | 9.9 | 0.15 ng/mL | 0.31 - 160 ng/mL |
| ICAM-1 | 4.0 | 11.4 | 1.94 pg/mL | 1.94 - 32700 pg/mL |
| IFN-γ | 7.6 | 9.7 | 0.37 pg/mL | 0.37 - 938 pg/mL |
| IL-1a | 3.5 | 3.9 | 0.09 pg/mL | 0.09 - 278 pg/mL |
| IL-1b | 1.7 | 4.8 | 0.05 pg/mL | 0.05 - 375 pg/mL |
| IL-2 | 14.9 | 13.0 | 0.09 pg/mL | 0.09 - 938 pg/mL |
| IL-4 | 11.5 | 20.0 | 0.02 pg/mL | 0.02 - 158 pg/mL |
| IL-5 | 2.5 | 9.2 | 0.14 pg/mL | 0.14 - 562 pg/mL |
| IL-6 | 10.0 | 14.5 | 0.06 pg/mL | 0.06 - 488 pg/mL |
| IL-7 | 17.0 | 15.1 | 0.12 pg/mL | 0.12 - 563 pg/mL |
| IL-8 | 7.9 | 6.4 | 0.07 pg/mL | 0.07 - 375 pg/mL |
| IL-10 | 19.0 | 21.5 | 0.04 pg/mL | 0.04 - 233 pg/mL |
| IL-12/IL-23p40 | 12.9 | 18.3 | 0.33 pg/mL | 0.33 - 2250 pg/mL |
| IL-12p70 | 17.6 | 22.3 | 0.11 pg/mL | 0.11 - 315 pg/mL |
| IL-13 | 13.7 | 17.5 | 0.24 pg/mL | 0.24 - 353 pg/mL |
| IL-15 | 6.0 | 9.4 | 0.15 pg/mL | 0.15 - 525 pg/mL |
| IL-16 | 1.7 | 3.9 | 2.83 pg/mL | 2.83 - 1870 pg/mL |
| IL-17a | 11.0 | 11.2 | 0.31 pg/mL | 0.31 - 3650 pg/mL |
| IP-10 | 8.7 | 12.3 | 0.37 pg/mL | 0.37 - 500 pg/mL |
| MCP-1 | 3.2 | 7.9 | 0.09 pg/mL | 0.09 - 375 pg/mL |
| MCP-4 | 14.0 | 21.7 | 0.18 pg/mL | 0.18 - 472 pg/mL |
| MDC | 1.8 | 9.7 | 1.22 pg/mL | 1.22 - 3700 pg/mL |
| MIP-1a | 4.2 | 9.2 | 3.02 pg/mL | 3.02 - 743 pg/mL |
| MIP-1b | 6.6 | 18.7 | 0.17 pg/mL | 0.17 - 520 pg/mL |
| SAA | 5.9 | 7.5 | 10.9 pg/mL | 10.9 - 138000 pg/mL |
| TARC | 11.5 | 11.2 | 0.22 pg/mL | 0.22 - 1120 pg/mL |
| TNF-a | 10.1 | 11.4 | 0.04 pg/mL | 0.04 - 248 pg/mL |
| TNF-b | 8.7 | 10.9 | 0.08 pg/mL | 0.08 - 458 pg/mL |

**Caption:** Assay characteristics of the 31 inflammatory biomarkers are presented, including intra- and inter-assay coefficients of variation (%CV), lower limit of detection (LLOD), and dynamic range. CRP = c-reactive protein; HMGB-1 = biomarker high mobility group box 1; ICAM-1 = intercellular adhesion molecule 1; IFN-γ = interferon γ; IL = interleukin; IL-12/IL-23p40 = IL-12/IL-23 p40 protein; IL-12 p70 = IL-12 p70 protein; IP-10 = interferon gamma-induced protein 10; MCP = monocyte chemoattractant protein; MDC = macrophage-derived chemokine; MIP-1a = macrophage inflammatory protein 1a; SAA = serum amyloid A; TARC = thymus- and activation-regulated chemokine; TNF = tumor necrosis factor
