# Supplementary material for: Neuroinflammatory Biomarkers for Traumatic Brain Injury Diagnosis and Prognosis: A TRACK-TBI Pilot Study
Source: Neurotrauma Rep. 2023 Mar 24;4(1):171–83. doi: 10.1089/neur.2022.0060 (PMC10039275; doi:10.1089/neur.2022.0060)
Supplement: Supplemental data [file Suppl_TableS2.docx]

**Supplemental Table 2. Discriminability of 31 Inflammatory Biomarkers Across TBI Diagnosis and Outcome Categories**

| **Biomarker** | **Biomarker Class** | **TBI vs. HC** | **TBI vs. OC** | **GCS 3-12 vs. 13-15** | **CT+ vs. CT-** | **GOSE 1-4 vs. 5-8 at 3-months** | **GOSE<8 vs. =8 at 3-months** | **GOSE 1-4 vs. 5-8 at 6-months** | **GOSE<8 vs. =8 at 6-months** |
| --- | --- | --- | --- | --- | --- | --- | --- | --- | --- |
| CRP | Acute Phase Reactant | 0.656 (0.573-0.738) | 0.617 (0.518-0.717) | 0.726 (0.618-0.834) | 0.733 (0.652-0.813) | 0.687 (0.553-0.821) | 0.558 (0.453-0.662) | 0.688 (0.528-0.848) | 0.617 (0.507-0.727) |
| Eotaxin | Chemokine | 0.533 (0.413-0.653) | 0.627 (0.530-0.725) | 0.569 (0.458-0.680) | 0.566 (0.476-0.655) | 0.564 (0.429-0.699) | 0.560 (0.437-0.683) | 0.594 (0.459-0.728) | 0.560 (0.433-0.687) |
| Eotaxin-3 | Chemokine | 0.606 (0.496-0.716) | 0.601 (0.504-0.697) | 0.517 (0.406-0.629) | 0.650 (0.564-0.735) | 0.648 (0.518-0.777) | 0.615 (0.506-0.725) | 0.453 (0.303-0.604) | 0.538 (0.408-0.668) |
| HMGB-1 | Alarmin | 0.860 (0.802-0.919) | 0.835 (0.774-0.895) | 0.621 (0.504-0.738) | 0.640 (0.553-0.728) | 0.588 (0.459-0.717) | 0.548 (0.433-0.663) | 0.572 (0.431-0.713) | 0.557 (0.433-0.681) |
| ICAM-1 | Adhesion Response | 0.666 (0.572-0.761) | 0.455 (0.353-0.557) | 0.573 (0.466-0.679) | 0.560 (0.470-0.650) | 0.624 (0.504-0.744) | 0.556 (0.446-0.666) | 0.611 (0.482-0.740) | 0.608 (0.494-0.722) |
| IFN-γ | Cytokine | 0.549 (0.442-0.656) | 0.533 (0.422-0.644) | 0.549 (0.430-0.669) | 0.560 (0.470-0.650) | 0.455 (0.316-0.594) | 0.575 (0.464-0.687) | 0.442 (0.284-0.599) | 0.568 (0.450-0.685) |
| IL-1a | Cytokine | 0.559 (0.397-0.722) | 0.538 (0.417-0.659) | 0.502 (0.393-0.611) | 0.559 (0.470-0.649) | 0.597 (0.463-0.732) | 0.575 (0.459-0.692) | 0.485 (0.329-0.642) | 0.529 (0.400-0.657) |
| IL-1b | Cytokine | 0.613 (0.514-0.712) | 0.795 (0.729-0.860) | 0.597 (0.488-0.707) | 0.495 (0.405-0.585) | 0.562 (0.432-0.693) | 0.617 (0.503-0.731) | 0.575 (0.439-0.712) | 0.612 (0.488-0.736) |
| IL-2 | Cytokine | 0.541 (0.405-0.677) | 0.602 (0.508-0.697) | 0.695 (0.585-0.804) | 0.663 (0.579-0.746) | 0.705 (0.587-0.823) | 0.542 (0.429-0.656) | 0.694 (0.566-0.821) | 0.498 (0.382-0.614) |
| IL-4 | Cytokine | 0.819 (0.731-0.907) | 0.580 (0.471-0.690) | 0.594 (0.490-0.699) | 0.590 (0.501-0.679) | 0.570 (0.439-0.702) | 0.524 (0.408-0.640) | 0.572 (0.432-0.712) | 0.488 (0.360-0.616) |
| IL-5 | Cytokine | 0.748 (0.621-0.874) | 0.674 (0.556-0.791) | 0.552 (0.436-0.669) | 0.598 (0.510-0.686) | 0.518 (0.380-0.655) | 0.529 (0.419-0.638) | 0.552 (0.407-0.697) | 0.581 (0.465-0.697) |
| IL-6 | Cytokine | 0.924 (0.880-0.967) | 0.533 (0.425-0.641) | 0.747 (0.650-0.844) | 0.757 (0.682-0.833) | 0.682 (0.549-0.814) | 0.602 (0.490-0.713) | 0.662 (0.509-0.814) | 0.617 (0.497-0.737) |
| IL-7 | Cytokine | 0.764 (0.637-0.891) | 0.742 (0.651-0.833) | 0.476 (0.371-0.580) | 0.526 (0.435-0.617) | 0.475 (0.352-0.598) | 0.549 (0.432-0.665) | 0.504 (0.361-0.648) | 0.543 (0.423-0.664) |
| IL-8 | Cytokine | 0.764 (0.666-0.862) | 0.689 (0.597-0.782) | 0.625 (0.521-0.728) | 0.667 (0.582-0.751) | 0.637 (0.527-0.747) | 0.602 (0.487-0.716) | 0.639 (0.526-0.751) | 0.591 (0.468-0.715) |
| IL-10 | Cytokine | 0.863 (0.804-0.922) | 0.668 (0.575-0.761) | 0.687 (0.574-0.799) | 0.634 (0.548-0.721) | 0.689 (0.564-0.813) | 0.541 (0.422-0.659) | 0.686 (0.551-0.822) | 0.614 (0.489-0.740) |
| IL-12/IL-23p40 | Cytokine | 0.622 (0.513-0.730) | 0.517 (0.406-0.628) | 0.637 (0.527-0.747) | 0.580 (0.491-0.669) | 0.550 (0.406-0.695) | 0.597 (0.489-0.705) | 0.596 (0.449-0.744) | 0.484 (0.363-0.605) |
| IL-12p70 | Cytokine | 0.675 (0.573-0.777) | 0.516 (0.402-0.630) | 0.488 (0.375-0.600) | 0.549 (0.459-0.639) | 0.488 (0.361-0.614) | 0.542 (0.428-0.657) | 0.517 (0.378-0.656) | 0.499 (0.379-0.619) |
| IL-13 | Cytokine | 0.676 (0.574-0.777) | 0.516 (0.402-0.631) | 0.488 (0.375-0.601) | 0.549 (0.459-0.639) | 0.488 (0.361-0.615) | 0.542 (0.428-0.657) | 0.516 (0.377-0.655) | 0.499 (0.379-0.619) |
| IL-15 | Cytokine | 0.659 (0.574-0.744) | 0.560 (0.468-0.653) | 0.720 (0.607-0.833) | 0.724 (0.644-0.804) | 0.738 (0.615-0.861) | 0.650 (0.539-0.761) | 0.704 (0.557-0.850) | 0.711 (0.607-0.815) |
| IL-16 | Cytokine | 0.727 (0.642-0.813) | 0.784 (0.709-0.858) | 0.668 (0.563-0.773) | 0.599 (0.510-0.687) | 0.614 (0.491-0.736) | 0.648 (0.540-0.757) | 0.642 (0.517-0.767) | 0.587 (0.472-0.701) |
| IL-17a | Cytokine | 0.541 (0.408-0.674) | 0.525 (0.398-0.652) | 0.579 (0.470-0.687) | 0.538 (0.448-0.628) | 0.423 (0.288-0.557) | 0.571 (0.457-0.684) | 0.502 (0.367-0.638) | 0.525 (0.402-0.647) |
| IP-10 | Chemokine | 0.619 (0.511-0.727) | 0.525 (0.420-0.629) | 0.550 (0.427-0.674) | 0.514 (0.424-0.604) | 0.588 (0.450-0.727) | 0.541 (0.426-0.656) | 0.594 (0.436-0.751) | 0.636 (0.526-0.747) |
| MCP-1 | Chemokine | 0.592 (0.463-0.721) | 0.487 (0.376-0.598) | 0.646 (0.538-0.754) | 0.611 (0.523-0.698) | 0.641 (0.526-0.755) | 0.573 (0.458-0.688) | 0.610 (0.464-0.756) | 0.632 (0.521-0.744) |
| MCP-4 | Chemokine | 0.645 (0.494-0.795) | 0.688 (0.585-0.792) | 0.597 (0.489-0.705) | 0.545 (0.454-0.636) | 0.486 (0.357-0.614) | 0.548 (0.432-0.664) | 0.643 (0.514-0.772) | 0.618 (0.492-0.744) |
| MDC | Chemokine | 0.680 (0.550-0.809) | 0.641 (0.535-0.747) | 0.592 (0.492-0.693) | 0.484 (0.393-0.574) | 0.598 (0.471-0.724) | 0.507 (0.395-0.618) | 0.593 (0.481-0.705) | 0.559 (0.447-0.671) |
| MIP-1a | Chemokine | 0.577 (0.438-0.715) | 0.584 (0.468-0.700) | 0.504 (0.388-0.620) | 0.608 (0.519-0.697) | 0.574 (0.438-0.711) | 0.526 (0.417-0.635) | 0.549 (0.390-0.707) | 0.455 (0.330-0.581) |
| MIP-1b | Chemokine | 0.588 (0.470-0.707) | 0.511 (0.412-0.610) | 0.647 (0.548-0.746) | 0.640 (0.553-0.727) | 0.639 (0.503-0.776) | 0.523 (0.395-0.650) | 0.681 (0.542-0.820) | 0.649 (0.529-0.768) |
| SAA | Acute Phase Reactant | 0.693 (0.597-0.789) | 0.581 (0.484-0.678) | 0.716 (0.608-0.825) | 0.767 (0.693-0.840) | 0.690 (0.561-0.820) | 0.567 (0.460-0.674) | 0.704 (0.564-0.843) | 0.603 (0.489-0.718) |
| TARC | Chemokine | 0.749 (0.626-0.872) | 0.731 (0.637-0.825) | 0.656 (0.557-0.756) | 0.531 (0.441-0.621) | 0.491 (0.362-0.620) | 0.561 (0.452-0.670) | 0.539 (0.408-0.669) | 0.552 (0.433-0.671) |
| TNF-a | Cytokine | 0.674 (0.573-0.775) | 0.516 (0.402-0.630) | 0.488 (0.375-0.600) | 0.550 (0.460-0.640) | 0.488 (0.361-0.615) | 0.543 (0.428-0.657) | 0.516 (0.377-0.655) | 0.500 (0.380-0.620) |
| TNF-b | Cytokine | 0.518 (0.374-0.662) | 0.508 (0.398-0.618) | 0.518 (0.403-0.633) | 0.579 (0.490-0.668) | 0.589 (0.451-0.727) | 0.527 (0.416-0.638) | 0.596 (0.450-0.742) | 0.499 (0.366-0.633) |

**Caption:** Discriminability is shown for all 31 biomarkers across TBI diagnosis and severity categories, and 3- and 6-month outcome categories. Biomarkers shaded in gray have acceptable discrimination within their respective categories (AUC >0.7; 95% confidence intervals shown). AUC = area under the receiver-operating characteristic curve; CRP = c-reactive protein; CT = computed tomography; GCS = Glasgow Coma Scale; GOSE = Glasgow Outcome Scale-Extended; HC = healthy control; HMGB-1 = biomarker high mobility group box 1; ICAM-1 = intercellular adhesion molecule 1; IFN-γ = interferon γ; IL = interleukin; IL-12/IL-23p40 = IL-12/IL-23 p40 protein; IL-12 p70 = IL-12 p70 protein; IP-10 = interferon gamma-induced protein 10; MCP = monocyte chemoattractant protein; MDC = macrophage-derived chemokine; MIP-1a = macrophage inflammatory protein 1a; OC = orthopedic control; SAA = serum amyloid A; TARC = thymus- and activation-regulated chemokine; TBI = traumatic brain injury; TNF = tumor necrosis factor
